# Simulating Net Ecosystem Productivity (NEP) in Mediterranean Pine Forests (*Pinus brutia*) During the 21st Century: The Effect of Leaf Area Index and Elevation

**DOI:** 10.3390/plants14071090

**Published:** 2025-04-01

**Authors:** Christodoulos I. Sazeides, Nikolaos M. Fyllas

**Affiliations:** 1 Biodiversity Conservation Laboratory, Department of Environment, University of the Aegean, 81100 Mytilene, Greece; 2 Section of Ecology and Taxonomy, Department of Biology, National & Kapodistrian University of Athens, 15772 Athens, Greece; nfyllas@biol.uoa.gr

**Keywords:** climate change, *Pinus brutia*, gross primary productivity, soil heterotrophic respiration

## Abstract

The Gross Primary Productivity (GPP) of Mediterranean forest is expected to change over the 21st century due to the warmer and drier conditions. In this study, we present a process-based forest carbon-flux model, where stand structure and soil heterotrophic respiration have been parameterized with long-term monitoring data in a Mediterranean *Pinus brutia.* Ten. forest. The developed model was validated using an independent annual tree-ring increment dataset from the 1980–2020 period (baseline climate) across a post-fire gradient (four plots) and an elevation gradient (five plots). Additionally, the model was forced with two downscaled climate change scenarios (RCP4.5 and RCP8.5) for the 2020–2100 period. Average GPP, Net Primary Productivity (NPP), ecosystem Respiration (R_eco_) and Net Ecosystem Productivity (NEP) were calculated for two future time periods (2051–2060 and 2091–2100) under the two climate change scenarios and compared along the two gradients. Under baseline climate conditions, our simulations suggest a temperature sensitivity of GPP and R_eco_, as expressed along the elevation gradient. However, the effect of stand structure (represented through the site-specific leaf area index (LAI)) was more prominent, both along the elevation gradient and the post-fire chronosequence. Under the two climate change scenarios, a reduced GPP and an increased R_eco_ lead to reduced NEP compared to baseline climate conditions across all study plots.

## 1. Introduction

Although forests at a global scale act as one of the main biospheric carbon (C) sinks, storing 2.4 ± 0.4 Pg C on an annual basis [1], some forest ecosystems may release C at specific temporal scales [2,3], and thus detailed accounting of the C-fluxes and understanding of the factors that control them is essential for designing adaptive forest management policies. The amount of C that is captured in any ecosystem per unit area and time is defined as the Net Ecosystem Productivity (NEP g C m^−2^ s^−1^), often calculated as the difference between Gross Primary Productivity (GPP g C m^−2^ s^−1^) and ecosystem Respiration (R_eco_ g C m^−2^ s^−1^). GPP represents the amount of C enchained by plants in the form of Carbon Dioxide (CO_2_) through photosynthesis. R_eco_ refers to the release of CO_2_ back to the atmosphere due to the respiration of all living organisms in the ecosystem. Two components of R_eco_ are usually identified: autotrophic respiration (R_aut_ g C m^−2^ s^−1^) and heterotrophic respiration (R_het_ g C m^−2^ s^−1^). The dynamic temporal and spatial balance between GPP and R_eco_ define whether an ecosystem acts as a sink or source of C [4].

Understanding the dynamics of the above ecosystem fluxes is crucial for modeling and projecting the role of forests in the global carbon cycle under current and future environmental conditions [5]. The rate and annual variability of photosynthetic activity is considered as the primary control of GPP. Environmental factors such as solar radiation [6], air temperature [7] and soil water content [8] are known to regulate the rate of photosynthesis at both the leaf and the canopy levels. In Mediterranean ecosystems, water stress can limit photosynthesis [9]. Experimental studies on Mediterranean plants have shown that the maximum rate of photosynthesis is achieved at lower temperatures than the theoretical optimum, when plants are under water stress, helping to avoid further water loss [10]. Stomatal closure, in response to water stress, limits the amount of CO_2_ uptake as well as the internal diffusion of CO_2_ from the substomatal cavities to the chloroplast, leading to lower photosynthetic rates [11] during periods of limited water availability. Even though photosynthetic metabolism is minimally impacted at stomatal conductance (Gs) above 0.1–0.15 mol H_2_O m^−2^ s^−1^, plants in the Mediterranean region often exhibit Gs lower than this threshold [10]. After such a severe event, plants require time to recover their photosynthetic rates, ranging from a day [12] to weeks, or even months [13], after the first rainfall event. Conversely, plants under Mediterranean climatic conditions exhibit higher temperature thresholds for tissue necrosis and lower inhibition of photosynthesis under high temperatures compared to plants in other regions [14,15]. The above underlines that accurate simulations of ecosystem dynamics should consider the potential adaptations that plants have developed, particularly at local scales and environmental conditions, highlighting the importance of regional scale process-based models that are parameterized with empirical monitoring data [6].

Plant autotrophic respiration is frequently modeled as the sum of maintenance and growth respiration [16,17]. An approach in process-based models is to estimate growth respiration as a fraction of plant Net Primary Productivity (NPP), and maintenance respiration as a function of biotic and abiotic factors [17]. However, R_aut_ is also empirically approximated through a linear relationship with plant size [18], challenging the dependence of maintenance respiration on abiotic factors. For example, a cross-biome analysis of belowground R_aut_ observations suggests that abiotic factors do not directly control R_aut_, but rather GPP, which when reduced, leads to lower plant respiration [19]. Following this line of logic, GPP could be considered a major driver of R_aut_. Analysis of C-fluxes from different biomes (from boreal forests to tropical) shows that R_aut_ varies from 40 to 65% of GPP, with the lower values in the Mediterranean biome [20].

Soil heterotrophic respiration (R_het_ g C m^−2^ s^−1^), which is the outcome of microbial and fungal activity, is mainly controlled by temperature and soil water content [21]. Temperature can directly affect microbial activity by changing the activation energy of their enzymes, as well as indirectly by limiting other factors, such as soil moisture [22]. When soil water content (θ cm^3^ cm^−3^) is low, R_het_ is limited due to microorganisms’ reduced conductivity to substrate that damages the organisms [23] and limited diffusion of the extracellular enzymes that break down the organic C [24]. However, in Mediterranean ecosystems that frequently experience a pronounced inter- and intra-annual climate variability, a linear reduction of R_het_ due to low θ may not occur, as rapid adaptations of the microbial community have been reported [25]. Although there is limited information on the response and recovery time of the soil microbial community after rehydration, a R_het_ pulse after rainfall events has been frequently reported in Mediterranean ecosystems [26,27]. On the other hand, excessive soil water content seems to inhibit soil respiration due to limitations in soil aeration [28,29]. Empirical soil respiration models developed for temperate and Mediterranean forests [26] often identify temperature, temperature variability, soil C and water content as the primary drivers of soil respiration. However, these parameters illustrate significant site to site variability and thus local studies and site-specific models could provide more accurate performance [30,31].

Pine forests are an important element of natural vegetation in areas surrounding the Mediterranean basin. At lower elevations (up to 600 m above sea level—asl), the two pine species *Pinus halepensis* and *Pinus brutia* are distributed in around 7 million hectares along the Mediterranean coastline [32]. At higher elevations (up to 2000 m asl), *Pinus nigra* covers 3.5 million hectares of the forested area [33]. Both forest types provide a range of ecosystem services and are estimated to absorb ca. 1586 and 1169 g C m^−2^ year^−1^, respectively [34,35]. However, under current climate change conditions and shifting fire regimes, the C sinks of these ecosystems might be at risk [36,37] and thus it is important to understand how shifting climate conditions might affect their C dynamics. According to the Intergovernmental Panel on Climate Change (IPCC) Fifth Assessment emission scenarios [38], the Mediterranean region is expected to experience rising average air temperatures (from 0.9 to 5.6 °C), reduced annual precipitation (from 4 to 22%) and more frequent drought events [39]. For example, climate projections based on the IPCC’s moderate RCP4.5 scenario suggest an average temperature increase of 2–2.5 °C for the eastern part of the Mediterranean, followed by up to 20% annual precipitation reductions until the end of the 21st century [40]. Projections based on the more pessimistic RCP8.5 scenario lead to a drier future, with 10–20% less precipitation and higher (5–6 °C) average temperatures by the end of the century [41]. In this latter trajectory, apart from the expected overall precipitation decrease, the frequency of extreme events is also projected to increase, leading to an overall rise in the number of dry days in the area [42].

In this study, we present the development, evaluation and application of a process-based forest C-flux model under different climate scenarios during the 21st century, aiming to explore whether stands of typical Mediterranean pine forests could remain carbon sinks or shift to carbon sources. The model is an extension of a previously published process-based model [43], with updated photosynthesis and a new respiration algorithm. We initially parameterized the model using monthly C-flux measurements in four Mediterranean pine forest stands (dominated by *Pinus brutia*) that represent a leaf area index (LAI—m^2^ m^−2^) trajectory along a post-fire chronosequence. We evaluated the model performance using a tree-ring dataset for the four (4) study plots and an additional group of five (5) *Pinus brutia* plots located along an elevation gradient on the island of Lesvos, Greece. We thus initially set up our process-based model to simulate the primary productivity of the nine (9) study plots for forty years (1980–2019), and its outputs were compared against the tree-ring width index extracted from the tree-ring dataset. Subsequently, the process-based model was used to simulate GPP, NPP and NEP during the 21st century (2000–2100), following the RCP4.5 and RCP8.5 climate scenarios. These outputs were used to explore the effect of stand structure and elevation on the productivity of Mediterranean pine forests under global warming conditions.

## 2. Materials and Methods

### 2.1. Study Sites

All study plots were located on the island of Lesvos, Greece, and were part of the permanent forest-plot monitoring network maintained by the Department of Environment, University of the Aegean. The climate of the island is characterised by long xero-thermic periods and mild wet winters. The dry season begins in mid-April and ends in early October. The average annual temperature on the island is approximately 18.0 °C, with the minimum monthly temperature in January (6.8 °C) and the maximum monthly temperature in mid-July (31.0 °C). Mean total annual precipitation is 645 mm, with most rainfall occurring in December and January (Hellenic National Meteorological Service) and a spatial variation from 725 mm in the eastern part to 415 mm in the western part of the island [44].

**Figure 1:**
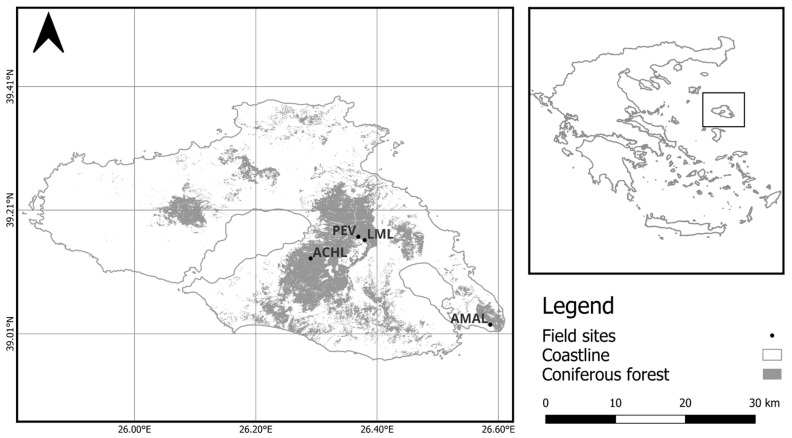
Distribution of the four plots with different amounts of time since the last stand-replacing fire on the island of Lesvos. The dominant tree species in all plots was *P. brutia*. See Table 1 for additional plot information. The coordinate system is WGS 84.

In this study, we used data from nine plots (four post-fire and five elevation gradient plots). In four of the study plots, tree-growth/mortality and stand structure data have been recorded since their establishment in early 2019 (Figure 1). These plots were established across a post-fire chronosequence [43] at the central-eastern part of the island, where forests are dominated by *Pinus brutia* (Table 1). The time since the last stand-replacing fire ranged from 13 to 92 years, with all the plots having similar elevations (166 to 316 m asl), mild slopes (under 10%) and soil parental material (Ophiolite). All four plots appeared to experience similar climatic conditions, with their long-term growth patterns well synchronized [45]. In these plots, soil respiration measurements were taken on a monthly basis.

**Figure 2:**
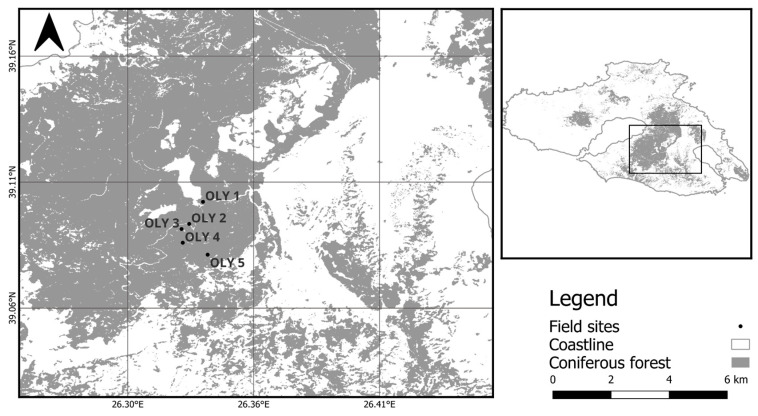
Distribution of the five plots located across the elevation gradient (from 350 to 750 m asl) on the island of Lesvos. The dominant tree species in all plots was *P. brutia*. See Table 1 for additional plot information. The coordinate system is WGS 84.

Five additional permanent monitoring plots, also dominated by *P. brutia* and established across an elevation gradient, were used in this study for model validation purposes. These plots are located on the central part of the island of Lesvos (Mount Olympos, Figure 2) at elevations ranging from 350 to 750 m asl, with a sequential elevation difference of 100 m. In these plots, we monitored only biometric parameters related to the structure of the stand, with long-term tree growth data also available from the analysis of annual tree-rings [45]. All five plots were established on similar parental material (Ophiolite) with a soil depth range from 20 to 40 cm (Table A3).

In each of the 9 study plots, air temperature and relative humidity were recorded for a period of almost three years, using iButton Hygrochron DS1923-F5# (iButtonLink, Whitewater, WI, USA), with a 5 h time step. All sensors were placed on a tree within the plot (at a height of 2 m) facing north. In a subset of the plots (5), soil temperature at 15 cm was also recorded. These data were used to calculate the daily mean, minimum and maximum air and soil temperature, and the average air RH, between July 2020 and July 2023. Across plots, mean annual temperature (T_A_) varied between 17.4 and 12.6 °C, with mean annual relative humidity (RH_A_) between 62.49 and 79.84% (Table 2). The daily average air temperature (T_mean_) data were used to estimate the local lapse rate (−0.638 °C/100 m), which was used to correct for temperature differences between plots, while a linear relationship between daily average soil temperature (T_s_) and air temperature was also developed.

### 2.2. Stand Structure and Tree Measurements

Within each one of the nine (9) permanent monitoring plots (30 × 30 m square), all trees above 1.3 m were identified to the species level and received a unique code to facilitate biometric measurements. For each tree, diameter at breast height (dbh—cm) and height (H—m) were measured in July 2019 and monitored every second year. The status (alive or dead) of each tree was also recorded since the start of the plot monitoring period. Soil samples were taken for texture and chemical analysis, and the mean soil depth was estimated for each plot, using an iron stick that was hammered until it hit the bedrock at five different points within the 900 m^2^ square.

The LAI of each plot was estimated using a ceptometer (Decagon Devices, Inc., Pullman, WA, USA) by systematically measuring photosynthetic active radiation (PAR) at 36 points above the forest floor (1.3 m) and comparing each PAR with that measured outside of the canopy under full light conditions. All LAI measurements were made around the solar noon, and the leaf distribution parameter was set to X = 1.

Long-term tree growth data were available from the analysis of increment cores that were extracted from at least 12 trees adjacent to the plots [45]. From each tree, two cores were extracted to minimize the possible sign from reaction wood. Cores were collected at breast height with the use of a 5 mm increment borer. After surface preparation, tree-ring width was measured to 0.01 mm using the Time Series Analysis and Presentation software (TSAP-Win version 4.81) [46] and a LINTAB (Rinntech Inc., Heidelberg, Germany) measuring table. All samples were visually and statistically cross-dated using TSAP-Win to identify the possible presence of missing or false rings [47]. For more details about the methodology, see [45]. Annual tree-growth data were used to calculate a plot-specific tree-ring width index for model comparison [6,48].

### 2.3. Gas Exchange Measurements

From July 2019 to December 2020, we monitored soil autotrophic and heterotrophic respiration. At each plot, we installed four PVC collars, each with 10 cm internal diameter and 10 cm height. Three of those collars were inserted at least 2 cm, directly into the soil, to measure the total soil respiration (Rs). To measure soil heterotrophic respiration, we dug a hole (approximate 30 cm wide and 40 cm deep), and the soil was extracted and separated into its characteristic layers. A bucket with a drainage hole was inserted to provide a barrier for the roots and refilled with the extracted soil layer by layer [49]. Old root fragments were excluded from the bucket. The fourth collar was inserted at least 2 cm into the bucket and left for a month to reach an equilibrium before the first measurement. This method yielded estimates of R_het_ and, by combining R_s_ and R_het_ measurements, we were able to estimate soil autotrophic respiration (R_aut_):

(1) $$R_{s}=R_{het}+R_{aut}$$

Soil respiration measurements were carried out using an LI-8100 Automated Soil CO_2_ Flux System (LICOR Biosciences Inc., Lincoln, NE, USA) infrared gas analyzer with a 10 cm survey chamber. For each collar, soil respiration was measured from early in the morning until the afternoon of each sampling day (3 to 5 measurements) to account for diurnal temperature and moisture fluctuations, with three replications per measurement. Soil respiration data were processed using the SoilFluxPro 4.2.1 software (LI-COR Biosciences, Inc., NE, USA). During each replicate, a minimum of 80 logs were made (one per second—max 105 logs), with this data used to fit an exponential curve. The slope of the curve was used to estimate R_het_ per replicate and the average slope was recorded as the per measurement R_het_.

At the time of each soil respiration measurement, soil temperature (T_s,i_) and water content (θ_i_) were recorded using the Omega 88311K thermometer (Omega Sensing Solutions ULC, Saint Eustache, QC, Canada) and the ML2x Theta Probe soil moisture sensor (Delta-T Devices Ltd., Burwell, Cambridge, United Kingdom) mounted on the LI-8100. The average values of T_s,i_ and θ_i_ during each measurement were used in the development of empirical R_het_ models (see R_het_ parametrization below).

### 2.4. Model Description

A full model description is provided in Appendix A. In the following section, we provide the main equations and their parameterization as implemented in the current model version.

#### 2.4.1. Primary Productivity

The simulation of daily canopy GPP (g C m^−2^ day^−1^) was based on the P-model v1.0 [50] and is given in Equation (2).

(2) $$GPP=\varphi_{0}\left(T\right)I_{abs}REWm^{\prime }Mc$$

where φ_0_ is the temperature-dependent intrinsic quantum yield efficiency (Equation (A2)), I_abs_ is the fraction of the PAR absorbed by the canopy (Equation (A3)) [51], REW is the soil relative extractable water by the plants (Appendix A, soil moisture section), *m*′ is the CO_2_ limitation factor for light-limited assimilation (Equation (A4)), and Mc is the molar mass of carbon (g mol^−1^).

NPP (g C m^−2^ day^−1^) was calculated by modifying the equation of DeLucia et al. [52]:

(3) NPP=0.53 GPP+(66.05/365)

assuming that a fraction of GPP (ca 0.47) is used for the whole plant autotrophic respiration (both above and below ground). The constant term in Equation (3) was estimated after converting the annual value to daily.

NEP (g C m^−2^ day^−1^) was estimated daily by subtracting R_het_ from NPP, assuming that the aboveground heterotrophic respiration of the ecosystem is a very small fraction of the total R_het_.

(4) $$NEP=NPP-R_{het}$$

R_het_ was parameterized by fitting an empirical mixed-effect model to the average daily available measurements (See Section 2.4.3).

#### 2.4.2. Soil Water Balance

A simple daily soil water balance model was used in the current version of the model, which kept track of the available soil water (AW—mm):

(5) $$AW=min\left\{\max\left\{{AW}^{*}+P-\left({REW}^{*}{PET}^{*}\right),{AW}_{r}\right\},{AW}_{fc}\right\}$$

where AW* is the available water the previous day, P (mm) is the amount of precipitation of the current day, REW* is the relative extractable water REW (mm) of the previous day and PET* (mm) is the potential evapotranspiration of the day before. AW_r_ and AW_fc_ refer to the residual available water and field capacity available water, respectively. Residual water is a certain amount of water that cannot be drained from the soil, even at high tension, due to it being retained in disconnected pores and immobile films [53]. The residual soil water content (AW_r_) and water content at field capacity (AW_fc_) were calculated based on the soil texture classification and the parameters provided in [54,55] for European soils (Appendix A, Table A1).

Daily soil water content (θ—m^3^ water m^−3^ soil) was calculated based on:

(6) $$\theta =(AW\;\rho_{W)}/\left(\rho_{soil}\;SD\right)$$

with ρ_Soil_ (g cm^−3^) the soil bulk density, ρ_W_ (g cm^−3^) the density of the water and SD the soil depth (mm).

The Campbell [56] water retention model was used to convert between soil water content and water potential:

(7) $$\Psi =-\Psi_{e}*{\left(\theta /\theta_{s}\right)}^{-b}$$

where Ψ_e_ (MPa) is the air-entry potential, θ_s_ the maximum volumetric soil water content at saturation and b the soil water retention parameter related to soil texture (see Appendix A, Table A1), with values per soil texture class taken from [54]. In order to account for the species differences in their ability to extract water from relatively dry soils, a species-specific minimum water potential was used when estimating REW:

(8) $$REW=\left(\theta -\theta_{Spmin}\right)/\left(\theta_{fc}-\theta_{Spmin}\right)$$

with θ_fc_ the soil water content at field capacity and θ_Spmin_ the soil water content corresponding to the minimum species-specific water potential Ψ_min_. For the study species, we used a Ψ_min_ value of 3.3 (MPa) [57].

The daily potential evapotranspiration rate was estimated using an empirical equation for Greece [58]:

(9) $$PET=\left(0.06257R_{ex}\right)/\left(1-0.0234T_{mean}\right)$$

with R_ex_ the extraterrestrial radiation (MJ m^−2^ day^−1^), calculated according to the FAO equation [59] and the average air temperature (in °C).

#### 2.4.3. Soil Respiration

Soil respiration is frequently modelled as a temperature-dependent function [60,61], but it is also known to be limited under low and high soil water contents [62]. In Mediterranean ecosystems, abrupt and large soil CO_2_ pulses have been observed after dry soil rewetting [27]. In that sense, empirical models that include both temperature and soil water content have been developed to better predict site-specific Rs [30,31]. In this study, we tested twelve different empirical models and used the one that better predicted R_het_ to simulate the daily heterotrophic CO_2_ soil flux (Appendix A, Table A2). We applied a linear mixed-effect modelling procedure, using plot as a random effect and soil temperature (T_s_), soil water content (θ) and leaf area index (LAI) as fixed effects. LAI was used as a proxy for soil organic content assuming that denser canopies would lead to higher SOC in the soil. The optimal model accounted for 58.7% (marginal) of the observed R_het_ variation and yielded the following equation:

(10) $$R_{het}=\exp\left(-4.31+0.25\;T_{s}-0.004\;{T_{s}}^{2}+5.36\;\theta -6.37\;\theta^{2}+0.69\;log\left(LAI\right)\right)$$

Statistical analyses and graphs were performed using the lme4 [63], lmerTest [64], sjPlot [65] and ggplot2 [66] packages.

### 2.5. Environmental Forcing Data

Daily climate data from the Lesvos airport weather station (Hellenic National Meteorological Service) were used to force the model for the 1980–2020 model evaluation period. The forcing data included T_mean_, T_min_, T_max_, RH_mean_, RH_min_, RH_max_, Precipitation, Radiation and CO_2_ concentration.

The daily simulated climate data from the Coordinated Downscaling Experiment—European Domain (EURO-CORDEX) were extracted for the region of interest at 0.11° resolution and used to force over the 21st century. We used the downscaled outputs of the MPI-ESM-LR Global Circulation Model [67,68] that provide continuous daily climate outputs for the periods 1970–2005 and 2006–2100, following the RCP4.5 and RCP8.5 emissions scenarios. The daily 1970–2005 climate data were compared with the data from the Lesvos airport weather station to derive a site-specific elevation (Z—m asl)-driven temperature correction equation:

(11) $$T_{mean}=T_{GCM}-(0.638*z/100)$$

with T_mean_ being the daily corrected average temperature and TGCM the temperature from the downscaled daily temperature from the MPI-ESM-LR general circulation model. The 0.638 coefficient represents the local lapse rate.

### 2.6. Model Evaluation and Simulations

The ability of our process-based model to predict NPP was evaluated against a tree-ring width index, extracted from the increment cores, from the 1980–2020 period. Our process-based model was also used to simulate carbon fluxes during the same period, forced with the climate dataset from the Lesvos airport. In each plot, a constant LAI was used (i.e., regeneration, growth and mortality were not considered). The simulated NPP from the process-based model was then compared with the simulated annual tree-ring width index, using a Pearson correlation analysis. As diameter tree-growth indices are related to NPP [69], we assumed that a linear association between the tree-growth index and our simulated NPP would indicate an agreement with an independent primary productivity estimation method. At this point, we note that we compared two independent data flows from the same site, i.e., annual tree-rings and process-based estimations in the case of our model.

To disentangle the effect of elevation (i.e., temperature) and leaf area index variation on primary productivity across the study sites, we conducted a simulation experiment where each factor was kept constant and simulation outputs were comparatively considered. The model setups were implemented. The conLAIvarT setup controlled for the effect of stand LAI variation by setting a constant LAI value (average across plots, 1.96 m^2^ m^−2^) for each plot and allowing temperature to vary based on the elevation of each plot. The varLAIconT setup controlled for the effect of temperature by setting the seasonal variation of temperature as identical for all plots (as per the 550 m asl plot) and setting the LAI specific value to be the one measured in the field. The varLAIvarT setup represented the most realistic model parameterization (the one used in the model evaluation exercise), where both LAI and temperature were allowed to vary based on the observed variation across the elevation gradient. The outputs of these simulations were comparatively considered to test which of the first two setups was closer to our most realistic parameterization.

The process-based model was additionally used to simulate carbon fluxes over the course of the 21st century following a baseline (current climate), an RCP4.5 and an RCP8.5 scenario. In this study, we mainly focused on the GPP, R_het_ and NEP model simulations over three reference periods: baseline (from 1996 to 2005), forced with the current climate conditions; mid-term (from 2051 to 2060, referred to also as 2060), forced with both the RCP4.5 and RCP8.5 climate scenarios; and long-term (from 2091 to 2100, referred to also as 2100), forced with both the RCP4.5 and RCP8.5 climate scenarios.

## 3. Results

### 3.1. Model Evaluation

**Figure 3:**
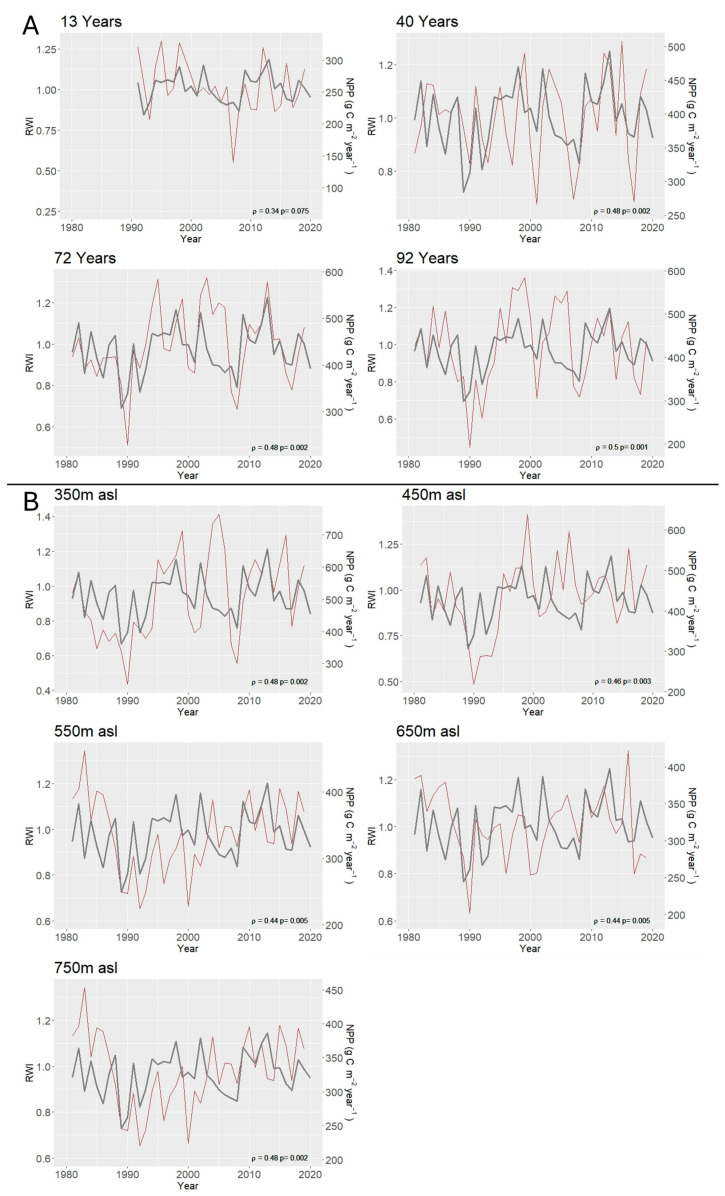
Interannual variation of simulated Net Primary Productivity (NPP) (from process-based model—grey line) and tree-ring width index (RWI—red line) during the 1980–2020 evaluation period. In the case of the younger plot (13 years), the period of the analysis was shorter. (**A**) Interannual NPP and RWI variation across the *P. brutia* post-fire chronosequence gradient. (**B**) Interannual NPP and RWI variation across the *P. brutia* elevation (350–750 m asl) gradient. Correlation coefficient (ρ) between NPP and RWI, as well as *p*-value (*p*), are indicated for each plot.

The intercomparison between the annual NPP process-based model output and the tree-ring width index indicated agreement across the post-fire chronosequence gradient with loose positive (up to ρ = 0.5) but statistically significant correlations, except for the younger plot (Figure 3). Along the elevation gradient plots, correlations between NPP and the tree-ring width index were positive but weaker (up to ρ = 0.48), with all being statistically significant (Figure 3).

### 3.2. Simulation Experiment to Explore the Effects of LAI, Air Temperature and Increased CO_2_

**Figure 4:**
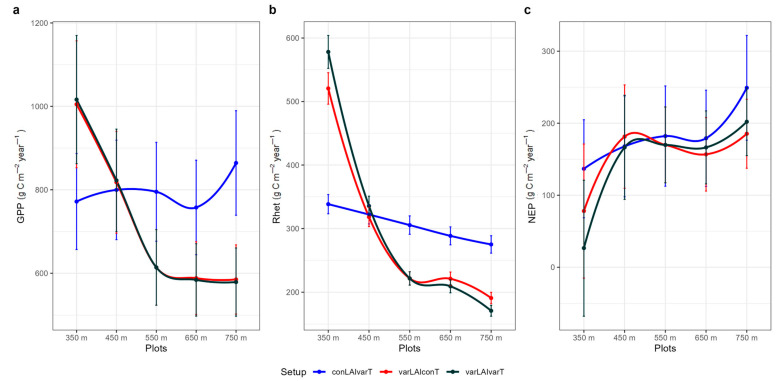
Estimated (**a**) GPP (Gross Primary Productivity), (**b**) R_het_ (soil heterotrophic respiration) and (**c**) NEP (Net Ecosystem Productivity) per elevation, and setup for elevation gradient plots. Setup conLAIvarT was with constant value for LAI (1.96) and variant temperature according to local lapse rate; varLAIconT was with variant LAI, according to our measurements, and constant temperature; and varLAIvarT was with variant LAI and temperature. Error bars are ± standard deviation.

Across the elevation gradient, the magnitude of the GPP flux was primarily controlled by the underlying LAI gradient, i.e., decreasing LAI with elevation (Table 1—OLY plots). The varLAIconT setup, which kept the temperature constant, provided identical outputs to the varLAIvarT setup, suggesting that the higher GPP at lower elevation was driven by the higher LAI of lower elevation plots rather than by their higher temperature (Figure 4a). A similar model behavior was observed for R_het_ and NEP, with LAI again having a stronger effect than temperature (Figure 4b,c).

### 3.3. Seasonal Variation of C-Fluxes Under Current and Future Conditions

**Figure 5:**
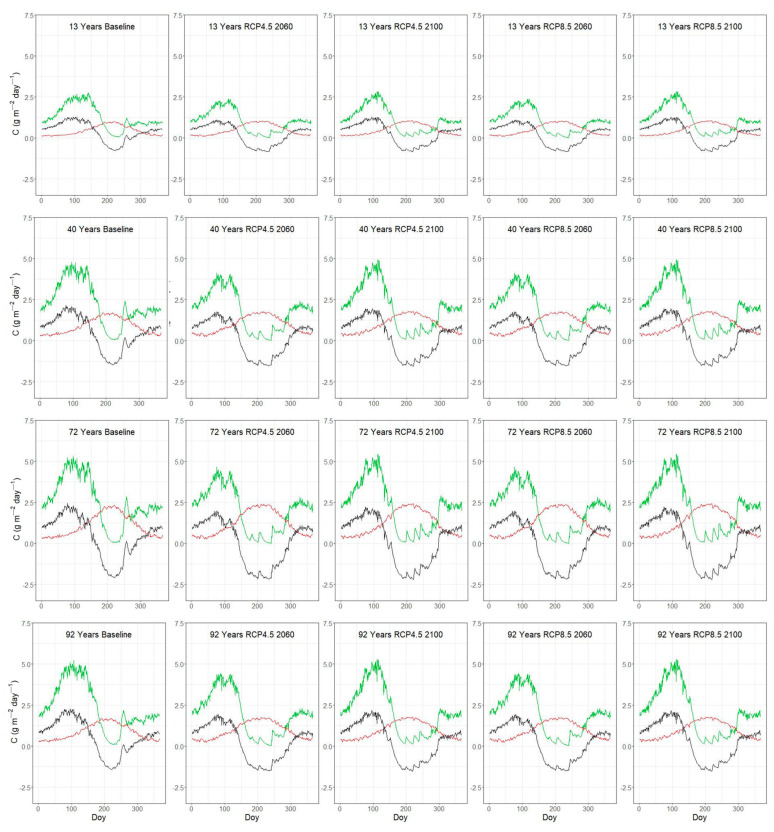
Simulated mean daily values of GPP (Gross Primary Productivity) as green line, R_het_ (soil heterotrophic respiration) as red line and NEP (Net Ecosystem Productivity) as black line for plots along the post-fire chronosequence gradient according to the day of the year (Doy).

**Figure 6:**
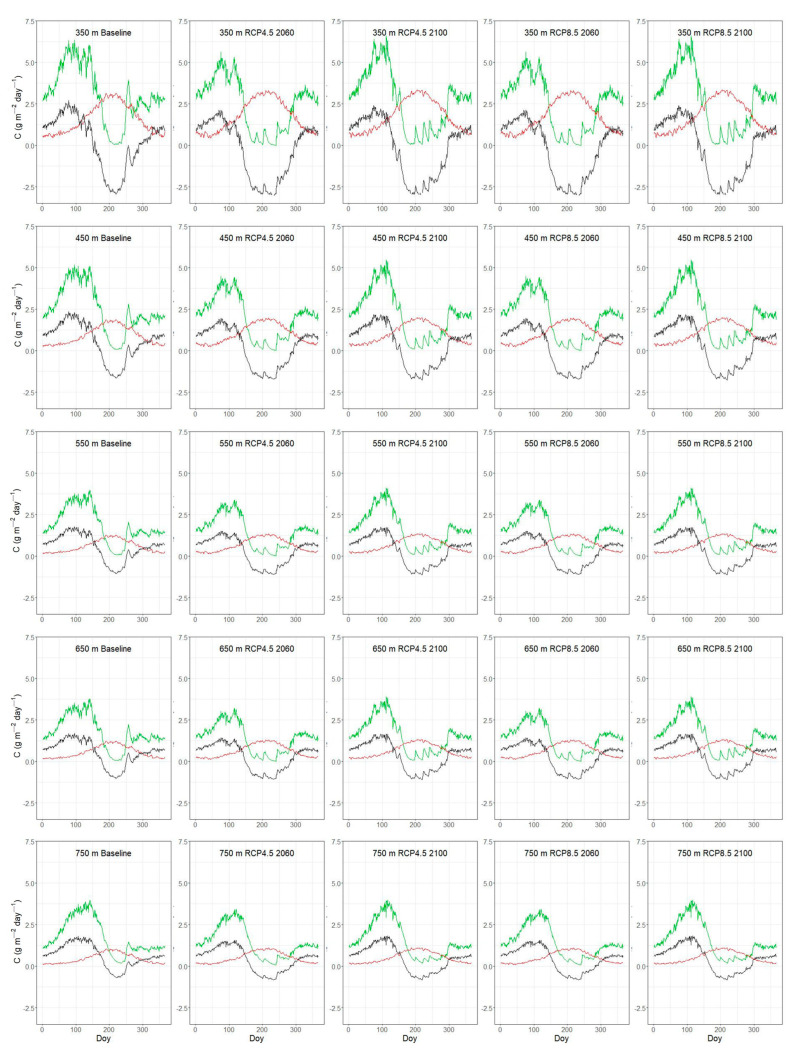
Simulated mean daily values of GPP (Gross Primary Productivity) as green line, R_het_ (soil heterotrophic respiration) as red line and NEP (Net Ecosystem Productivity) as black line for plots along the elevation gradient according to the day of the year (Doy).

The simulated daily average C-fluxes over the three study periods (1996–2005, 2051–2060 and 2091–2100) are illustrated in Figure 5 and Figure 6. The intra-annual C-flux patterns were similar across the *P. brutia* fire chronosequence plots (Figure 5), driven by incoming radiation and soil moisture variation, with GPP peaking during the spring period (doy: 100–150) and increasing again during autumn (doy: 250–300), with the NPP following the same pattern as GPP. R_het_ displayed a bell-shaped curve that peaks during the summer period (doy: 150–250), and NEP presented a sharp decline over the summer period with negative values mainly occurring at the same period (doy: 150–300). Under increasing climate change severity, the period of negative NEP was simulated to extend and reach more negative values.

Across the *P. brutia* elevation gradient, the intra-annual variation of C-fluxes followed a similar temporal pattern to GPP with NPP, increasing during spring, R_het_ peaking during summer and NEP declining to negative values over the summer (Figure 6). Comparing the trajectory of GPP and its seasonal patterns across different scenarios and periods (except the baseline), no particular differentiation was observed. However, the period of increased R_het_ extended with increasing scenario severity.

### 3.4. Annual C-Fluxes Under Current and Future Climate Conditions

**Figure 7:**
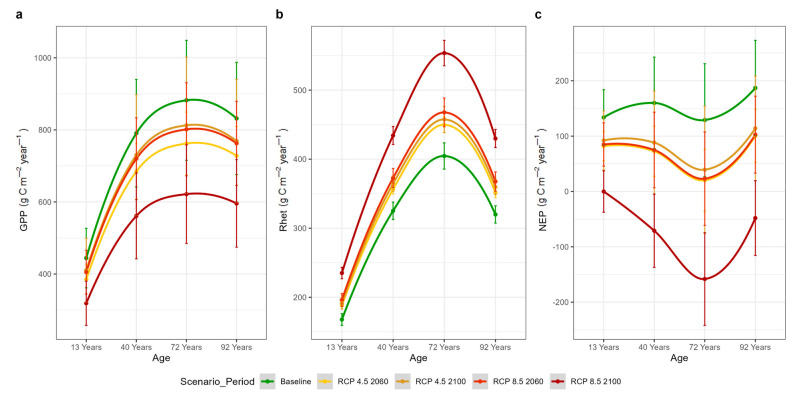
Estimated (**a**) GPP (Gross Primary Productivity), (**b**) R_het_ (soil heterotrophic respiration) and (**c**) NEP (Net Ecosystem Productivity) per stand age, scenario and period for the post-fire chronosequence plots. Error bars represent ± standard deviation during the baseline (1996–2005), mid-term (2051–2060, referred to as 2060) and long-term (2091–2100, referred to as 2100) periods per climate scenario.

Across the post-fire chronosequence plots, the effect of stand structure, as regulated by the time since the last fire event, on GPP and NPP was similar across all climate scenarios. In particular, GPP seemed to increase with stand age and LAI, from ca 445 to 880 g C m^−2^ year^−1^, and then to decline in the mature (92 years old) plot to ca 830 (g C m^−2^ year^−1^) under baseline climate conditions (Figure 7a). NPP followed a similar pattern to GPP, ranging from ca 300 to 530 g C m^−2^ year^−1^, with the mature stand reaching 505 g C m^−2^ year^−1^. This GPP vs. age trajectory remained consistent under both climate change scenarios and reference periods. Across all post-fire plots, simulated GPP was 7.7–13.8% and 8.8–28.8% lower than the baseline GPP under the RCP4.5 and RCP8.5 scenarios, respectively (Table 3).

A similar pattern was observed for R_het_ which also increased with stand age, from ca 170 to 400 g C m^−2^ year^−1^ before declining in the mature stand at ca 320 g C m^−2^ year^−1^ under baseline climate conditions (Figure 7b). Across the two climate change scenarios, R_het_ exhibited a greater percentage difference from baseline conditions, compared to GPP (Table 3). Notably, under the RCP8.5 scenario in the last period, R_het_ was higher than in all other scenarios and periods.

In the post-fire chronosequence plots, NEP followed a different pattern. NEP was simulated to peak at the mature stand (92 years old), varying from ca 130 to 190 g C m^−2^ year^−1^ from the younger to the older plot, respectively, under baseline climate conditions (Figure 7c). Across all post-fire plots, the mean NEP under the different climate change scenarios ranged from 46.1 to 148% of baseline conditions (Table 3), with the RCP8.5 scenario during the last period showing the lowest values compared to all other simulations.

**Figure 8:**
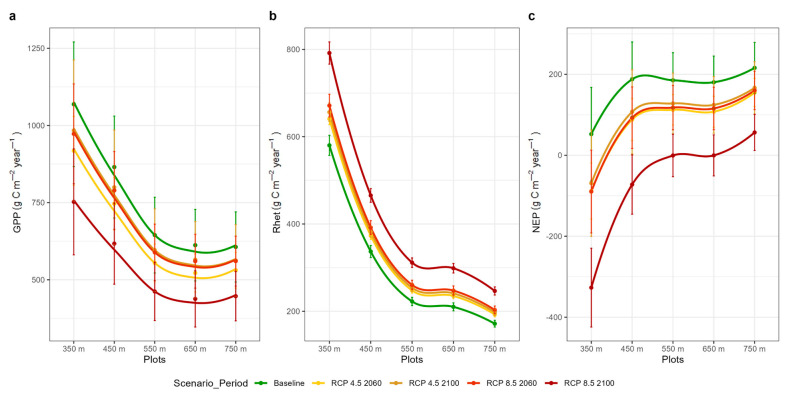
Estimated (**a**) GPP (Gross Primary Productivity), (**b**) R_het_ (soil heterotrophic respiration) and (**c**) NEP (Net Ecosystem Productivity) per elevation, scenario and reference period. Error bars represent ± standard deviation during the baseline (1996–2005), mid-term (2051–2060, referred to as 2060) and long-term (2091–2100, referred to as 2100) periods per climate scenario.

The annual GPP of plots found across the elevation gradient ranged from ca 605 to 1070 g C m^−2^ year^−1^ under baseline conditions (Figure 8a) and decreased with the severity of the climate change scenario (Table 3). At the same time, NPP was estimated to range from ca 390 to 630 g C m^−2^ year^−1^. Under baseline conditions, R_het_ ranged from ca 170 to 580 g C m^−2^ year^−1^ and NEP from ca 50 to 220 g C m^−2^ year^−1^. R_het_ increased with climate change severity, while NEP decreased (Table 4).

Overall, across all study plots and climate conditions, GPP showed lower variation and greater stability compared to R_het_. The stronger R_het_ changes yielded a decrease in Net Ecosystem Productivity (Table 3 and Table 4). Under the RCP8.5 scenario for the 2100 period the strongest ecosystem-level C assimilation reduction was simulated.

## 4. Discussion

In this study, we developed a process-based C-flux model constrained with data from forest monitoring plots that simulates the GPP and the NEP of Mediterranean *P. brutia* stands. To do so, we used a set of field measurements to empirically parametrize parts of the model. After validating the model’s predictive ability across a post-fire chronosequence and elevation gradient, we simulated stand-level C-fluxes for two future periods (i.e., 2051–2060 and 2091–2100) and two climate change scenarios (RCP4.5 and RCP8.5). Our simulations suggest that LAI, considered here as a proxy of a stand’s structure and disturbance history, is the most important determinant of C-fluxes. Under climate change conditions and for the specific characteristics of our simulated plots, NEP remains positive, i.e., stands remain C-sinks for most future periods and climate scenarios, except in the case of the pessimistic RCP8.5 scenario in the longer-term 2091–2100 simulation period.

### 4.1. Model Verification

In this study, we tested the predictive ability of our process-based model by comparing the time series of simulated NPP at each plot with available plot-specific annual tree-ring data. Previous studies have shown that estimated NPP does not necessarily show a one-to-one correlation with data from annual tree-rings, as the latter may be formed from both the current years’ assimilated carbon as well as from carbon stored from previous years [70,71]. Across our study sites, the Pearson correlation coefficient between the simulated NPP and the observed ring width index was always positive and ranged from 0.34 to 0.50, suggesting that the general patterns of interannual C-fluxes are captured by the process-based model. Other studies that have used annual tree-ring width indices to compare independent model NPP output for three forest types found statistically significant results only in two of them, with an R^2^ = 0.12–0.21 [72]. On the other hand, a study that used annual tree-ring data to estimate NPP found a statistically significant correlation between NPP with RWI, with ρ ranging from 0.109 to 0.804 from the smallest to the largest tree diameter class, respectively [69].

It should be noted that, as our model does not specifically simulate between-tree competition, recruitment and/or mortality, but rather uses a static stand structure, these results mainly verify the realism of the temperature and water availability effects on primary productivity. Thus, our model is suitable for simulating C-fluxes in stands with a known structure, which should be provided as a model input from either forest inventory or remote sensing data.

### 4.2. GPP

The seasonal variation of GPP (Figure 5 and Figure 6) is controlled by the seasonal dynamics of climatic variables such as radiation, temperature and precipitation [73]. The range of simulated GPP under baseline climate conditions is relatively lower compared to the values obtained for Mediterranean pine forests, for example, in Tuscany with a mean estimated value of 1760 g C m^−2^ year^−1^ [74], but closer to values reported in other modelling studies, ca 900 g C m^−2^ year^−1^ [34]. The analysis of eddy-flux tower data reports GPP values for Mediterranean pine forest in the range of 714–1151 g C m^−2^ year^−1^ [75]. With our simulations, we were able to explore the effect of stand LAI and elevation on the primary productivity of typical Mediterranean pine forest on the island of Lesvos. Under baseline climate conditions, GPP ranged from ca 445 to 880 g C m^−2^ year^−1^ across plots of varying LAI, determined by the time since the last stand-replacing fire. GPP followed the expected trajectory with stand age, increasing from younger to mature stands and then gradually decreasing following variation in LAI [43,76]. Simulations along our elevation (temperature) gradient plots, which are also characterized by decreasing LAI with elevation (Table 5), ranged from ca 605 to 1070 g C m^−2^ year^−1^ and revealed a stronger effect of LAI than temperature variation on GPP (Figure 4a). The above suggests that based on our model parameterization and the environmental conditions found across our study plots, a temperature difference of ca four degrees has a “weaker” effect on GPP than a ca four-unit difference in LAI. One study, based on a global dataset, highlighted temperature and LAI as important GPP drivers, among others, with mean annual temperature explaining 23% of the GPP variation and LAI explaining about 40% of the GPP variation [76].

Under climate change conditions, the simulated annual GPP was lower across all post-fire chronosequence plots, as well as the plots located along the elevation gradient (Figure 5 and Figure 6). These outputs suggest that photosynthetic water limitation could significantly reduce the assimilated carbon in Mediterranean pine forests. Our findings contradict simulations using the BIOME-BGC model, which predicted an increase in GPP for Mediterranean conifers following the RCP4.5 scenario [77], but do agree with the strong decrease in GPP projected for some Mediterranean conifer forests under the RCP8.5 scenario [78]. In general, our results show that across our study plots the potential positive effects of rising temperature and CO_2_ fertilization on photosynthetic carbon gain seem to be neutralized by enhanced water limitations [79,80].

### 4.3. Soil Heterotrophic Respiration

**Figure A1:**
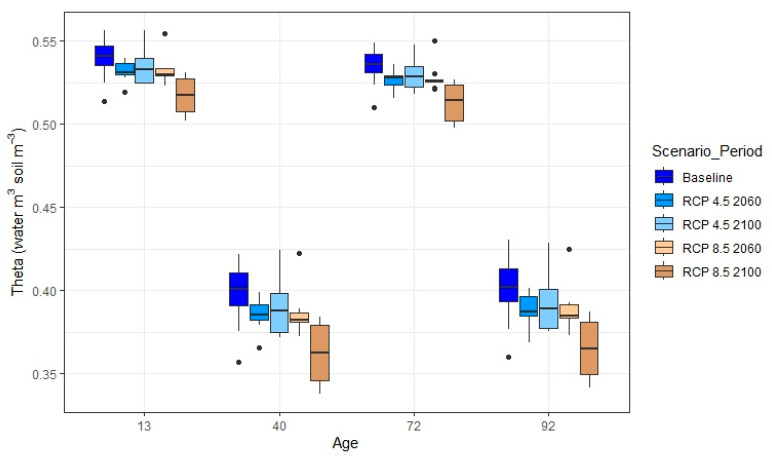
Estimated θ (soil volumetric water content) per plot, climatic scenario (RCP4.5 and RCP8.5) and period. Baseline period (1996–2005), 2060 period (2051–2060) and 2100 period (2091–2100).

Heterotrophic soil respiration is driven by variation in soil temperature [81,82], with the dynamics and interaction with the soil microbial community increasing the complexity of this fundamental ecosystem C-flux [83,84]. At the same time, the soil microbial community may shift to more heat-resistant (bacteria) or cold-resistant (fungi) species [85]. Such dynamic microbial community responses could change the expected slope of the R_het_–temperature relationship. A detailed analysis of different empirical soil respiration models [86] suggested that a Gaussian function enabling the inclusion of an optimum temperature at which R_het_ reaches its maximum best described a range of available soil respiration data. In our case, the empirical model used allowed for a decrease in R_het_ at high temperatures and soil moisture conditions (Equation (10)). At the same time, leaf area index was also used as a predictor of soil respiration, having in general positive effects on daily and monthly mean fluxes, and expressing the potential amount of easily available C to soil microbes [87]. Our empirical model also included a positive LAI effect on R_het_. Coupling the empirical soil heterotrophic respiration equation into the process-based model showed that, under all climate change scenarios, R_het_ increased (Figure 5, Figure 6, Figure 7 and Figure 8) regardless of the lower future soil moisture conditions (Figure A1). These outputs suggest that across our sites, under future conditions, soil moisture remains sufficient to maintain microbial/fungal activity, while the warmer conditions boost respiration [21,88]. Experiments simulating “warming only”, “drought only” and the “combination of conditions” demonstrated that R_het_ increased most under the “combination of conditions,” even with a 40% reduction in water. This increase was greater than that observed in the “warming only” or “drought only” scenarios, supporting our findings [89]. Moreover, our simulations highlight the sensitivity of R_het_ to changes in climate conditions compared to the greater robustness of GPP (Table 3 and Table 4). This suggests that R_het_ is more susceptible to shifts in temperature, precipitation and soil water availability, as soil microorganisms are more abundant in the upper soil layers [90] and are thus more exposed to variation in the local environmental conditions.

### 4.4. NEP

Under the baseline climate conditions, the simulated NEP (Table 5) in our study plots was always positive (i.e., stands act as C-sinks), in agreement with other field-based and modeling studies in Mediterranean ecosystems [74,91,92,93,94]. Evrendilek et al. [92] using inventory data estimated an average NEP for *P. brutia* stands in Turkey of around 320 g C m^−2^ year^−1^, a value slightly higher than the one simulated with our model.

According to our simulations under the two climate change scenarios, changes in NEP are primarily driven by variations in R_het_, which exhibit a larger percentage shift (Table 3 and Table 4) compared to GPP, except under the RCP4.5 scenario in the 2060 period. Overall, NEP is projected to decrease under all scenarios and study periods. Similar findings were reported for the Iberian Peninsula, where approximately 53% of the negative trends in NEP were attributed to negative trends in NPP and positive trends in R_het_ [95].

Theoretically, NEP is expected to be negative during the early years following a disturbance [96], due to the greater release of carbon through respiration than the photosynthetic C uptake. Following this initial phase, ecosystem productivity begins to exceed respiration, leading to positive NEP. Eventually, productivity and respiration reach a steady state, balancing NEP. In our case, the simulated NEP of post-fire plots was always positive but still followed this general trend. The younger (13 years old) plot appeared to have overcome the initial negative NEP phase. The oldest plot (92 years old) seemed to be approaching maximum productivity based on the shape of the curve during the baseline period (Figure 7). The parabolic trajectory was disrupted in the 72-year-old plot where NEP was lower than expected. Interestingly, a new trend emerged concerning the severity of climate scenario with. the 72-year-old plot showed stroger decrease in NEP compared to all other plots. This decline seemed to be primarily driven by the significant rise in R_het_, probably due to the contribution of several factors. Some of these may be the high soil moisture values (θ values) maintained by the “Very Fine” soil texture (Appendix A, Figure A1), the relatively warmer conditions in this low elevation plot and the relatively higher LAI. All these parameters can favor higher R_het_. Notably, under the extreme RCP8.5 2100 scenario and study period, all of our chronosequence plots shifted from C sinks to C sources and the overall trend of NEP seemed to reverse.

### 4.5. Model Limitations

The simulations presented in the current study were based on the big-leaf modelling methodology [97], where the canopy of a stand is considered a homogeneous layer with constant photosynthetic parameters. The photosynthetic algorithm used in the current model version (P-model v1.0, 2020) does not use species-specific parameters nor does it define different canopy layers (sun/shade) with discrete photosynthetic characteristics. It is thus a generic photosynthetic carbon assimilation algorithm that optimizes the carbon assimilation–transpiration trade-off. An alternative implementation of the sun/shade big-leaf model for the Mediterranean pine forests of Lesvos has been presented in Sazeides et al. [43] with GPP estimates similar to those presented in this study. A species-specific implementation of our model is currently under development.

In the current version of the model, a key addition was the empirical soil heterotrophic respiration algorithm, which is constrained with site-specific soil flux data. This enabled us to estimate NEP and explore whether the study plots will remain C-sinks under future climatic conditions. In that respect, a species- and (potentially) site-specific stand autotrophic respiration model could increase the confidence of our simulations. It should be noted that the current version of the model lacks demographics, i.e., it provides snapshots of annual carbon fluxes on predefined stands. Consequently, the model cannot be used for dynamic vegetation simulations but rather can be set up to simulate C-fluxes across stands with varying environmental conditions and stand structure.

## 5. Conclusions

Overall, this study provides a method to predict Net Ecosystem Productivity across Mediterranean pine forest stands. Despite its limitations, the model seems capable of realistically predicting GPP, NPP and NEP as well as their interannual variation. Across the post-fire chronosequence and the elevation gradient used in the current study, variation in LAI seems to have the strongest effect on C-fluxes, suggesting that stand structure should be specifically taken into account when estimating the carbon balance of Mediterranean pine forests. The application of the model under climate change conditions projected that, at least for most of the study plots on the island of Lesvos, *P. brutia* forests would probably retain their ability to store C, if the fire disturbance and the drought-related mortality regimes do not significantly alter their stand structure.

## Figures and Tables

**Table 1:** Geographic characteristics and stand age of the study plots. Latitude (Lat) and Longitude (Lon) are in decimal degrees. Stand age was estimated by counting the number of annual tree-rings in trees adjacent to the study plots [45]. Plots AMAL, PEV, LML and ACHL were located across a post-fire chronosequence, while OLY plots were located along an elevation gradient. Leaf area index (LAI) was estimated using a ceptometer.

Plot	Lat	Lon	Stand Age (y)	Elevation (m asl)	Inclination (%)	Orientation	LAI (m^2^ m^−2^)
AMAL	39.02	26.59	13	316	10	SE	0.67
PEV	39.16	26.37	40	213	5	SW	1.66
LML	39.16	26.38	72	166	10	SW	2.13
ACHL	39.13	26.30	92	261	0	-	1.67
OLY1	39.09	26.33	82	350	3	NE	4.26
OLY2	39.09	26.33	95	450	7	N	2.08
OLY3	39.09	26.33	96	550	5	N	1.23
OLY4	39.08	26.33	64	650	7	SE	1.23
OLY5	39.08	26.34	90	750	0	-	0.98

**Table 2:** Climatic description of the study plots corresponding to a three-year-long dataset as recorded within each plot. T_A_ is the annual average temperature, T_min_ and T_max_ are the annual average minimum and maximum temperatures, respectively. RH_A_ is the annual average relative humidity, RH_min_ and RH_max_ are the annual average minimum and maximum relative humidities, respectively. Also, T_sA_, T_smin_ and T_smax_ are the annual average, annual average minimum and annual average maximum soil temperatures. Temperatures are in °C and relative humidities are in percentage (%).

Plot	Elevation (m)	T_A_	T_min_	T_max_	RH_A_	RH_min_	RH_max_	T_sA_	T_smin_	T_smax_
AMAL	316	17.4	12.6	23.1	68.7	48.1	88.0			
PEV	213	17.3	10.6	25.4	67.2	38.5	92.6			
LML	166	16.6	9.2	24.7	70.8	44.8	91.5			
ACHL	261	17.3	10.8	26.9	66.6	36.8	88.9			
OLY1	350	16.5	10.3	25.8	62.5	41.5	78.7	15.8	14.3	17.6
OLY2	450	15.7	10.5	21.8	68.5	45.7	88.1	15.4	14.6	16.2
OLY3	550	15.4	10.4	21.6	69.6	47.1	90.0	14.9	14.1	15.7
OLY4	650	14.2	8.8	20.5	71.8	47.4	92.5	14.3	13.5	15.2
OLY5	750	12.6	8.5	18.3	76.0	59.5	88.1	12.3	11.6	13.0

**Table 3:** Mean percentage difference of annual GPP (Gross Primary Productivity, g C m^−2^ year^−1^), R_het_ (soil heterotrophic respiration, g C m^−2^ year^−1^) and NEP (Net Ecosystem Productivity, g C m^−2^ year^−1^), estimated per scenario and reference period compared to baseline conditions across all post-fire chronosequence plots. Column names refer to the climate change scenario (RCP4.5 or RCP8.5) and the period (2051–2060 or 2091–2100, referred to as 2060 or 2100, respectively).

	RCP4.5 2060	RCP4.5 2100	RCP8.5 2060	RCP8.5 2100
GPP (% change)	−13.2	−7.7	−8.8	−28.8
R_het_ (% change)	10.7	12.9	15.5	36.2
NEP (% change)	−56	−46.1	−54.2	−148

**Table 4:** Mean percentage difference in annual GPP (Gross Primary Productivity, g C m^−2^ year^−1^), R_het_ (soil heterotrophic respiration, g C m^−2^ year^−1^) and NEP (Net Ecosystem Productivity, g C m^−2^ year^−1^) estimates per scenario and reference period compared to baseline conditions across all plots along the elevation gradient. Column names refer to the climate change scenario (RCP4.5 or RCP8.5) and the period (2051–2060 or 2091–2100, referred to as 2060 or 2100, respectively).

	RCP4.5 2060	RCP4.5 2100	RCP8.5 2060	RCP8.5 2100
GPP (% change)	−13.8	−7.6	−8.5	−28.3
R_het_ (% change)	11.4	14.2	17	84
NEP (% change)	−86	−71.8	−52.3	−227.7

**Table 5:** Mean of annual NEP and standard deviation (SD) of estimations to baseline conditions across post-fire and elevation gradient plots.

Plots	NEP (g C m^−2^ s^−1^)	
	Mean	SD
13 Years	134	±50.0
40 Years	160	±82.7
72 Years	129	±101.8
92 Years	187	±86.0
350 m a.s.l.	52	±115.4
450 m a.s.l.	188	±92.2
550 m a.s.l.	185	±68.1
650 m a.s.l.	180	±64.6
750 m a.s.l.	216	±62.8

**Figure A2:**
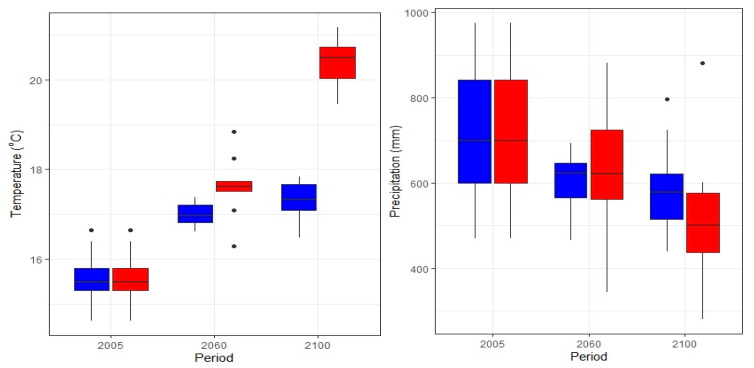
Temperature and precipitation boxplots for the baseline, 2060 and 2100 periods for RCP4.5 (blue) and RCP8.5 (red) scenarios.

## Data Availability

Data are available upon request from the first author.

## Appendix A

### Appendix A.1. Model Description

#### Appendix A.1.1. Primary Productivity

The simulation of daily canopy GPP is based on the P-model v1.0 [50], by multiplying with the stand’s LAI.

(A1) $$GPP=\varphi_{0}\left(T\right)I_{abs}REWm^{\prime }Mc$$

where φ_0_ is the temperature-dependent (T) intrinsic quantum yield efficiency, I_abs_ is the PAR absorbed by the canopy, REW is the soil relative extractable water by the plants (see Equation (A20)), m′ is the CO_2_ limitation factor for light-limited assimilation and Mc is the molar mass of carbon (g mol^−1^).

The temperature-dependent intrinsic quantum yield of photosynthesis is calculated from:

(A2) $$\varphi_{0}\left(T_{mean}\right)=c_{L}(0.352+0.022T_{mean}-0.00034{T_{mean}}^{2})$$

where c_L_ = 0.08718; it is a parameter determining the quantum efficiency of photosynthesis from leaf light absorptance [50].

The fraction of PAR that is absorbed by the canopy is given by:

(A3) $$I_{abs}=PPFD(1-\exp\left(-0.51LAI\right))$$

where PPFD is the sum of the PAR of the day.

The CO_2_ limitation factor for light-limited assimilation is given by:

(A4) $$m^{\prime }=m\sqrt{1-{\left(c^{*}/m\right)}^{2/3}}$$

with

(A5) $$m=\frac{C_{a}-\Gamma_{Τ}^{*}}{C_{a}+2\Gamma_{Τ}^{*}+3\Gamma_{Τ}^{*}\sqrt{\frac{1.6\eta^{*}D}{\beta (Κ+\Gamma_{Τ}^{*})}}}$$

c* is equal to 0.41 [98], Cα is the ambient CO_2_ concentration (Pa), D is the vapor pressure deficit, η* is the change in the viscosity of the water relative to its value at 25 °C (water viscosity at T°C/water viscosity at 25°C) and β is the unit cost ratio, taken as 146.0 [50]. For D, K and $\Gamma_{Τ}^{*}$, see Equations (A6), (A9) and (A13), respectively.

Water vapor pressure deficit D (Pa) is calculated by the difference of the pressure of water vapor in saturated air (esat) and the actual vapor pressure (eact) as

(A6) $$D=e_{sat}-e_{act}$$

with

(A7) $$e_{sat}=611\exp\left(17.27T/\left(T+237.3\right)\right)$$

Note that, if temperature data are not instant, but daily, e_sat_ is more desirable to calculate as the mean of e_sat_min_ and e_sat_max_ using the daily T_min_ and T_max_, respectively, and that T is in °C.

(A8) $$e_{act}=\frac{e_{sat\_min}\frac{{RH}_{max}}{100}+e_{sat\_max}\frac{{RH}_{min}}{100}}{2}$$

K is the Michaelis–Menten coefficient for photosynthesis (Pa) given by:

(A9) $$K\left(T_{K},p\right)=K_{c}\left(T_{K}\right)\left(1+(pO_{2}/K_{o}\left(T_{K}\right))\right)$$

with $K_{c}\left(T_{K}\right)$ and $K_{o}\left(T_{K}\right)$ being the Michaelis–Menten constants for carboxylation and oxygenation reactions, respectively (Pa):

(A10) $$K_{c}\left(T_{K}\right)=K_{c}*\exp({\Delta H}_{K_{c}}*(T_{K}-298.15)/(298.15*R*T_{K}))$$

(A11) $$K_{o}\left(T_{K}\right)=K_{o}*\exp({\Delta H}_{K_{o}}*(T_{K}-298.15)/(298.15*R*T_{K}))$$

where $K_{c}$ = 39.97 (Pa), $K_{o}$ = 27480 (KPa), ${\Delta H}_{K_{c}}$ = 79430 (J mol^−1^), ${\Delta H}_{K_{o}}$ = 36380 (J mol^−1^) and $T_{K}$ is the leaf temperature in °K. R is the universal gas constant (8.3145 J mol^−1^ K^−1^). pO_2_ is the partial pressure of oxygen in the atmosphere, corrected for elevation differences (z), with the assumption of a constant mixing ratio:

(A12) $$pO_{2}=0.2095\;p_{0}{\left(1-(Lz/T_{K,0})\right)}^{{gM_{a}(RL)}^{-1}}$$

with p_0_ the atmospheric pressure at sea level (Pa), L the mean adiabatic lapse rate (0.0065 °K m^−1^), z is the elevation (m), g the gravitational constant (9.80665 m s^−2^) and M_α_ is the molecular weight for dry air (0.028963 kg mol^−1^).

The photorespiratory compensation point is calculated from:

(A13) $$\Gamma_{Τ}^{*}=\Gamma^{*}*\exp({\Delta H}_{\Gamma *}*(T_{K}-{T_{K}}_{0})/({T_{K}}_{0}*R*T_{K}))$$

with ${\Delta H}_{\Gamma *}$ being the activation energy (23,400 Pa) and Γ* being the photorespiratory compensation point at 25 °C (4.332 Pa).

After the calculation of GPP, Net Primary Productivity is given by:

(A14) NPP=0.53 GPP+66.05/365

Net Ecosystem Productivity is calculated as:

(A15) $$NEP=NPP-R_{het}$$

For R_het_, see Table A2 (grey line). All GPP, NPP, NEP and R_het_ units are (g C m^−2^ day^−1^).

#### Appendix A.1.2. Soil Water Balance

(A16) $$AW=min\left\{\max\left\{{AW}^{*}+P-\left({REW}^{*}{PET}^{*}\right),{AW}_{r}\right\},{AW}_{fc}\right\}$$

with AW* (mm) the available water the day before (mm), P (mm) the daily precipitation, REW* the relative extractable water REW (mm) and PET* (mm) the potential evapotranspiration of the previous day. AW_r_ and AW_fc_ are the residual available water and field capacity available water, respectively.

AW is calculated as:

(A17) $$AW=\theta \left(\rho_{soil}/\rho_{W}\right)SD$$

with $\rho_{soil}$ the bulk density of the specific soil type, $\rho_{W}$ the water density and SD is the soil depth of the plot (see Table A1 for parameter values per soil texture type).

**Table A1:** Soil parameters [54] used in the soil moisture sub-model.

Soil Texture	Proportion of Clay and Sand	θ_r_	θ_s_	GMD	GSD
Coarse	18% < clay and >65% sand	0.025	0.403	0.4	5.3
Medium	18% < clay < 35% and ≥15% sand, or 18% < clay and 15% < sand < 65%	0.01	0.439	0.3	7.4
Medium Fine	<35% clay and <15% sand	0.01	0.430	0.1	6.1
Fine	35% < clay < 60%	0.01	0.520	0.07	14
Very Fine	Clay > 60%	0.01	0.614	0.007	15

The volumetric soil water content θ is estimated as:

(A18) $$\theta =\theta_{s}{\left(\Psi_{e}/\Psi_{i}\right)}^{-1/b}$$

with

(A19) $$\Psi_{e}=-0.5{GMD}^{-0.5}$$

(A20) $$b=-2\Psi_{e}+2.0GSD$$

θ_s_ is the maximum volumetric soil water content (see Table A1), when soil is totally saturated, Ψ_e_ (MPa) is the water potential at the maximum soil hydration, Ψ_i_ (MPa) is the minimum water potential and b is a function of soil texture.

Species-specific relative extractable water is estimated from:

(A21) $$REW=\left(\theta d-\theta_{Spmin}\right)/\left(\theta_{fc}-\theta_{Spmin}\right)$$

with

(A22) $$\theta_{d}=\left(AW\rho_{W}\right)/\left(\rho_{soil}SD\right)$$

and θ_fc_ the water content at field capacity (Ψ_fc_ = 0.033 MPa), θ_Spmin_ the water content for Ψ equal to the species-specific minimum water potential (Ψ_min_) and θ_d_ the average daily soil water content.

Finally, the daily potential evapotranspiration is estimated from an empirical equation for Greece [58]:

(A23) $$PET=\left(0.06257R_{ex}\right)/\left(1-0.0234T\right)$$

with R_ex_ (MJ m^−2^ day^−1^) the daily extraterrestrial radiation.

#### Appendix A.1.3. Soil Heterotrophic Respiration

Soil heterotrophic respiration has been incorporated in the process-based model by using an empirical equation fitted to the available monitoring data from the study sites. In particular, 12 empirical equations [99] that used average soil temperature (Ts), average soil moisture (θ) and leaf area index (LAI) as predictors were fitted and the one with the lowest AIC and highest marginal R2 was selected. The results of this analysis are summarized in Table A2, with the selected model highlighted in grey.

**Table A2:** Results of the linear mixed-effect model analysis. σ^2^ is the within group variance, τ_Plot_ the between plots variance, and ICC is the intraclass correlation coefficient. The marginal R^2^ is the variance attributed strictly to the fixed intercept and slopes, and the conditional R^2^ represents the variance attributed to both the fixed and random terms. The grey colored line indicate the equation which better describes our data, according to the marginal R^2^ and the Akaike Information Criterion, AIC.

Model	Random Effects			R^2^ Marginal	R^2^ Conditional	AIC
R_het_ =	σ^2^	τ_Plot_	ICC			
exp (Ts)	0.22	0.11	0.34	0.017	0.352	149
exp (T_s_ + θ)	0.21	0.14	0.40	0.045	0.424	147
exp (T_s_) × θ	0.21	0.14	0.40	0.040	0.421	152
exp (T_s_ + T_s_^2^)	0.15	0.09	0.39	0.239	0.536	128
exp (T_s_ + T_s_^2^ + θ)	0.10	0.16	0.61	0.328	0.738	100
exp (T_s_ + T_s_^2^) × θ	0.11	0.15	0.58	0.304	0.710	109
exp (T_s_ + T_s_^2^ + θ + θ × Τ_s_)	0.10	0.17	0.63	0.341	0.754	100
exp (T_s_ + T_s_^2^ + θ + θ^2^)	0.10	0.16	0.62	0.330	0.744	95
exp (T_s_ + T_s_^2^ + θ + θ × Τ_s_ + LAI)	0.10	0.06	0.36	0.555	0.716	99
exp (T_s_ + T_s_^2^ + θ + θ^2^ + LAI)	0.10	0.04	0.26	0.577	0.688	93
exp (T_s_ + T_s_^2^ + θ + θ × Τ_s_) × LAI	0.10	0.05	0.32	0.570	0.709	99
exp (T_s_ + T_s_^2^ + θ + θ^2^) × LAI	0.10	0.03	0.23	0.587	0.684	93

### Appendix A.2. Additional Information

**Table A3:** Plot-level parameter values across the study sites. LAI (leaf area index); Lat and Lon are the latitude and longitude, respectively.

	Plot	LAI (m^2^ m^−2^)	Soil Texture	Soil Depth (mm)	Elevation (m)
Post-fire gradient	AMAL	0.67	Very Fine	500	316
	PEV	1.66	Fine	270	213
	LML	2.13	Very Fine	300	166
	ACHL	1.67	Fine	350	261
Elevation gradient	OLY1	4.26	Fine	200	350
	OLY2	2.08	Fine	250	450
	OLY3	1.23	Fine	250	550
	OLY4	1.23	Fine	200	650
	OLY5	0.98	Fine	400	750

**Table A4:** Key model variables and their respective units.

Symbol	Description	Units
AW	Available soil water	mm
b	Function of soil texture	-
C_a_	Partial pressure of CO_2_ in the atmosphere	Pa
C_i_	Partial pressure of CO_2_ in the leaf	Pa
D	Vapor pressure deficit	Pa
e_act_	Actual vapor pressure	Pa
e_sat_	Pressure of water vapor in saturated air	Pa
GPP	Gross Primary Productivity	g C m^−2^ day^−1^
I_abs_	Fraction of PAR absorbed by the canopy	umol quanta m^−2^ day^−1^
K	Michaelis–Menten coefficient for photosynthesis	Pa
m′	CO_2_ limitation factor for light-limited assimilation and electron transport capacity	-
m	CO_2_ limitation factor for light-limited assimilation	-
NEP	Net Ecosystem Productivity	g C m^−2^ day^−1^
NPP	Net Primary Productivity	g C m^−2^ day^−1^
R_aut_	Soil autotrophic respiration	mol CO_2_ m^−2^ day^−1^
REW	Relative extractable water	-
R_ex_	Extraterrestrial radiation	MJ m^−2^ day^−1^
R_het_	Soil heterotrophic respiration	mol CO_2_ m^−2^ day^−1^
T_K_	Temperature	°K
Γ*_Τ_	Temperature-dependent photorespiratory compensation point	Pa
η*	Change in the viscosity of the water, relative to its value at 25 °C	-
θ	Soil water content	cm^3^ cm^−3^
θ_fc_	Soil water content at field capacity	cm^3^ cm^−3^
θ_s_	Water content of water-saturated soil	cm^3^ cm^−3^
θ_wp_	Soil water content at wilting point	cm^3^ cm^−3^
φ_0_	Intrinsic quantum yield efficiency	mol mol^−1^
Ψ_e_	Water potential at the maximum soil hydration	MPa
Ψ_i_	Water potential	MPa

**Table A5:** Model constants.

Symbol	Description	Units	Value
c*	Proportional to the unit carbon cost for the maintenance of electron transport	-	0.41
c_L_	Photosynthesis quantum efficiency parameter	-	0.08718
g	Gravitation constant	m s^−2^	9.80665
K_c_	Michaelis–Menten constants for carboxylation reactions	Pa	39.97
K_o_	Michaelis–Menten constants for oxygenation reactions	Pa	27,480
L	Adiabatic lapse rate	°K m^−1^	0.0065
Mc	Molecular mass of carbon	g mol^−1^	12.0107
M_α_	Molecular mass of dry air	kg mol^−1^	0.028963
p_0_	Atmospheric pressure at sea level	Pa	101,325
pO_2_	Partial pressure of oxygen in the atmosphere at sea level	Pa	21,000
R	Universal gas constant	J mol^−1^ K^−1^	8.3145
$\rho_{W}$	Density of the water	g cm^−3^	0.9982
β	Unit cost ratio	-	146
Γ*	Photorespiratory compensation point at 25 °C	Pa	4.332
ΔH_Kc_	Activation energy for Kc	J mol^−1^	79,430
ΔH_Ko_	Activation energy for K_o_	J mol^−1^	36,380
ΔH_Γ*_	Activation energy for Γ*	Pa, J mol^−1^	23,400
